# Subclavian steal syndrome associated with right aortic arch: A case report

**DOI:** 10.3389/fsurg.2022.1063224

**Published:** 2023-01-06

**Authors:** Jing Cai, Chao Xie, Xianwei Wang

**Affiliations:** ^1^ Department of General Practice, Xiangya Hospital, Central South University, Changsha, China; ^2^ Department of Cardiovascular Surgery, The Second Xiangya Hospital, Central South University, Changsha, China; ^3^ Department of General Surgery, Xiangya Hospital, Central South University, Changsha, China

**Keywords:** right aortic arch, subclavian steal syndrome, bypass surgery treatment, case, report

## Abstract

The right aortic arch (RAA) is a rare congenital vascular variant disease. We reported a case of subclavian steal syndrome associated with RAA. The primary clinical symptoms were vertigo and ischemic symptoms of the left upper extremity. We diagnosed the condition using aortic computed tomography angiography and digital subtraction angiography. The patient underwent carotid-subclavian bypass surgery.

## Case presentation

**Figure 1:**
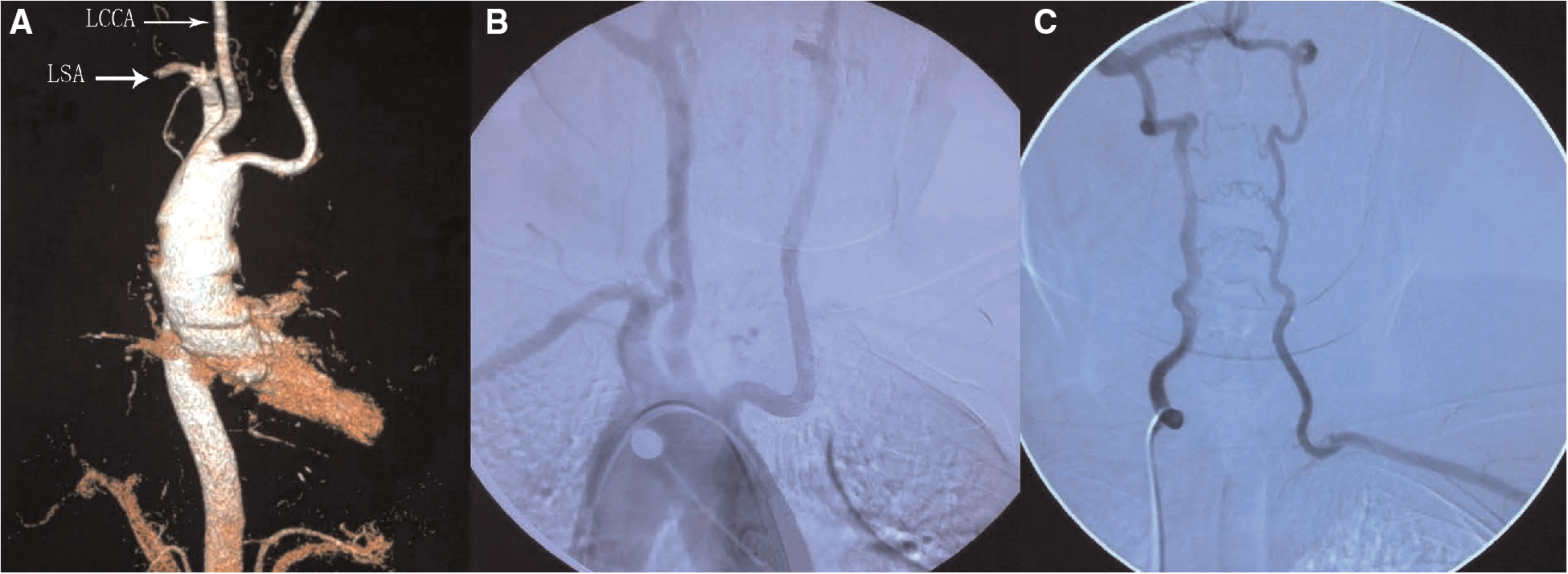
Preoperative CTA of the aorta showed a right-sided aortic arch with an abnormal great branch (**A**). Aortic DSA showed that the left subclavian artery was not visible (**B**). Left subclavian artery supplied by the left vertebral artery (**C**). LSA, left subclavian artery; LCCA, left common carotid artery.

A 60-year-old female patient was admitted to the hospital after she reported experiencing frequent nausea and dizziness for more than 1 year. Vertigo did not reveal a significant relationship with the body position and spontaneously resolved after a while. For a year, the appeal symptoms had recurred. She presented with a 3-year history of hypertension and coronary heart disease. The patient's mother and three sisters had a history of identical undiagnosed vertigo. Upon admission, physical examination revealed that the blood pressures of the left upper limb and the right upper limb were 113/67 mmHg and 157/85 mmHg, respectively, and the systolic pressure difference between the two upper limbs was 44 mmHg. Although the right brachial and radial arteries were normal, the pulse rates of the left brachial and radial arteries were low. The left hand and forearm had slightly lower skin temperature than the right hand and forearm. The sensory muscle strength of both forearms was normal. The right aortic arch and aberrant great branch of the aortic arch were detected by thoracic aortic computed-tomography angiography (CTA) (Figure 1A). The left subclavian artery was not visible. as revealed by the aortic arc digital subtraction angiography (DSA) (Figure 1B). The left vertebral artery (LVA) supplied the left subclavian artery (LSA) (Figure 1C).

## Diagnosis and treatment

**Figure 2:**
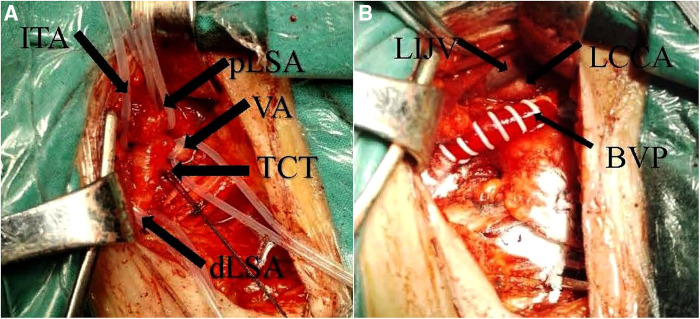
Intraoperative dissociation of the left subclavian artery and its branches, proximal narrowing of the lumen of the left subclavian artery is seen (**A**). Common carotid artery—left subclavian graft (**B**). PLSA, proximal left subclavian artery; DLSA, distal left subclavian artery; VA, vertebral artery; ITA, internal thoracic artery; TCT, thyroid trunk; BVP, artificial vessel; LCCA, left common carotid artery; LIJV, left internal jugular vein.

**Figure 3:**
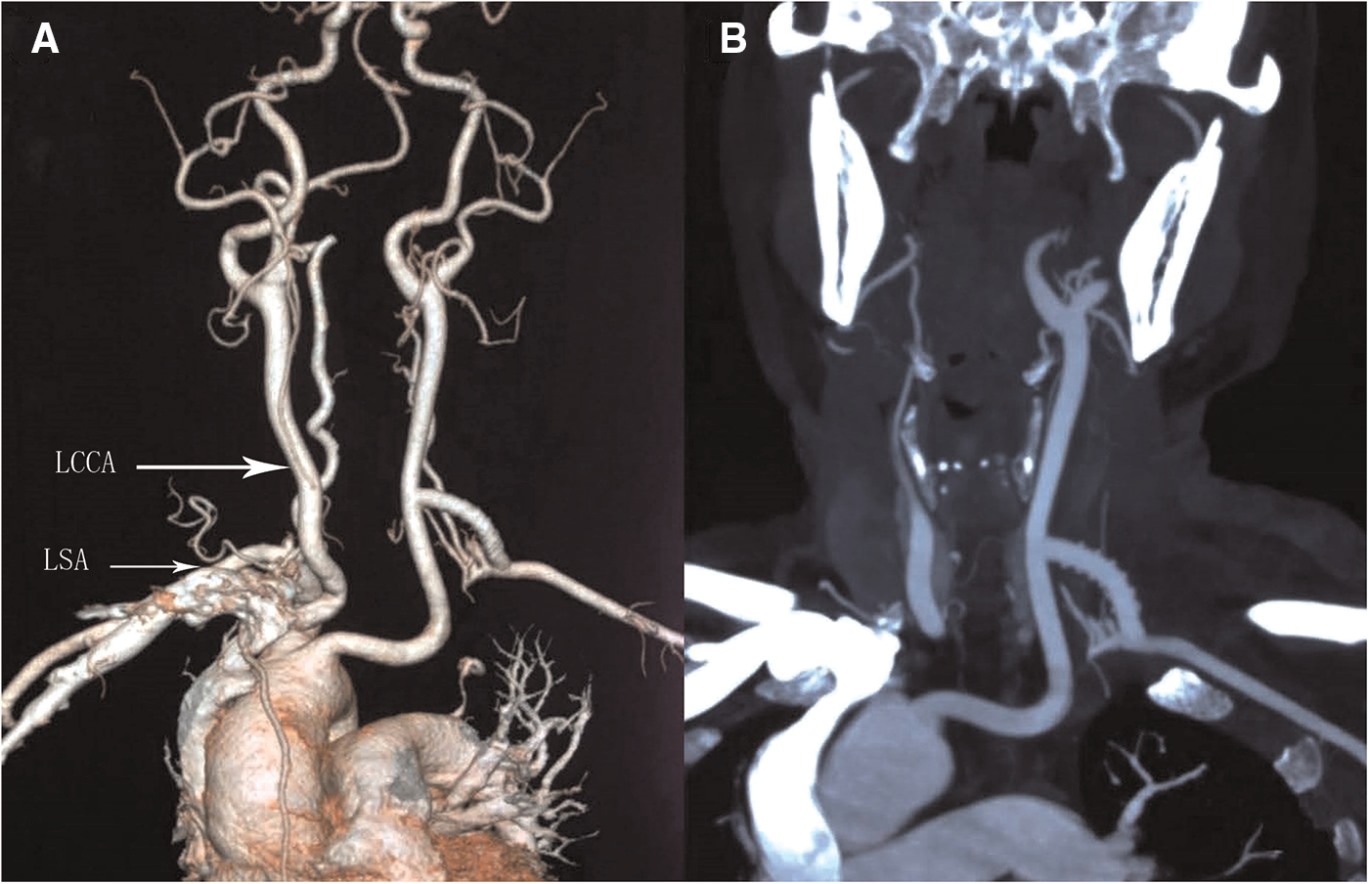
Postoperative CTA showed that good angiography of bypass vessels (**A,B**). LSA, left subclavian artery; LCCA, left common carotid artery.

The patient underwent a left common carotid artery-subclavian bypass surgery (Figure 2). The patient's vertigo improved following surgery. The throb of the left brachial artery and radial artery was intensified. The skin temperature of the left hand and forearm was elevated; however, there was no significant difference observed in the blood pressure of the two upper limbs. A postoperative review of cervical vascular CTA (Figure 3) revealed effective bypass vessel angiography. Our patient had no symptoms associated with subclavian steal syndrome at 3-month and 6-month follow-up.

## Discuss

With a global prevalence of 0.1%, RAA is a rare vascular malformation that can either be a mirror image of normal branches or a combination of various abnormalities (1, 2). Cases of subclavian steal syndrome associated with RAA are quite rare. One report showed symptoms of left arm ischemia and vertebrobasilar insufficiency years after left subclavian artery ligation (3). There are reports of a man with Peutz-Jeghers syndrome who had his first episode of vertigo at the age of 29 due to subclavian steal syndrome (4).

The ductus arteriosus is usually on the left side. However, the ductus arteriosus may also be present on the right side, possibly with bilateral arterial development associated with aortic arch malformations (3). The patient included in our study belonged to the third category. The separation of the left subclavian artery from the right aortic arch may be related to 22q11 deletion in embryological development (5, 6).

Contorni was the first to define subclavian steal syndrome, a condition in which blood flow is reversed in branches of the subclavian artery (7). The criteria for the diagnosis of subclavian steal syndrome are as follows: (1) Occlusion or marked stenosis of the subclavian or innominate arteries; (2) retrograde vertebral arterial flow; (3) patency of both the vertebral and basilar arteries (8). Our case met these criteria. Owing to the inadequate blood supply to the brain arteries, some patients with subclavian steal syndrome may experience symptoms of temporary ischemia (9). The symptoms of this disease include vertigo, dizziness, headache, diplopia, and a wide spectrum of symptoms involving the brain stem (1). It is caused by posterior circulation (brainstem vestibular system) ischemia, and vertigo generally lasts for several minutes and subsides on its own and thus can be distinguished from pseudovertigo, which is caused by hypertension (10). To accurately diagnose the subclavian syndrome, it is imperative to ascertain the blood pressure (BP) differences between retrograde blood flow in the arm and vertebral arteries (11, 12). More than 95% of patients have been documented in the literature to have overtly visible clinical symptoms, including dizziness, vomiting, headache, and syncope. The external carotid artery, the thyrocervical artery, the external carotid artery-occipital artery, the muscular branches of the vertebral artery systems, and a crossover flow between the internal mammary arteries are all collateral pathways for blood to enter the subclavian artery; however, the main anastomotic network is the vertebrobasilar system (10).

The primary imaging tests used to diagnose this disease were transthoracic echocardiography (TTE), magnetic resonance imaging (MRI), and angiography. Eighty-four percent of the cases were suspected based on the initial TTE; however, in many cases, echocardiography often indicated a suspicion albeit did not confirm the diagnosis. To determine the details of vascular connections, further imaging is generally required. In 38% of the cases, cardiac catheterization was used, allowing for hemodynamic assessment. A cardiac CT scan is preferred if the airway needs to be evaluated (13). Moreover, compared to other modalities, a cardiac CT scan can detect a smaller or stenotic patent ductus arteriosus (14). MRI provides the advantage of assessing blood flow through vessels with small diameters and into the heart chambers (15).

The patient underwent left common carotid artery-subclavian bypass surgery, which reduced the stress on the vertebral artery by diverting blood flow from the left common carotid artery to the subclavian artery. The patient's vertigo improved following surgery. The throb of the left brachial artery and radial artery was enhanced. The bypass surgery treatment proved to be an effective method of surgery for the patient.

## Data Availability

The original contributions presented in the study are included in the article/Supplementary Material, further inquiries can be directed to the corresponding author/s.
